# Development of a Summarized Health Index (SHI) for Use in Predicting Survival in Sea Turtles

**DOI:** 10.1371/journal.pone.0120796

**Published:** 2015-03-24

**Authors:** Tsung-Hsien Li, Chao-Chin Chang, I-Jiunn Cheng, Suen-Chuain Lin

**Affiliations:** 1 National Museum of Marine Biology and Aquarium, Checheng, Pingtung, 94450, Taiwan; 2 Graduate Institute of Microbiology and Public Health, National Chung Hsing University, Taichung, 40227, Taiwan; 3 Institute of Marine Biology, National Taiwan Ocean University, Keelung, 20224, Taiwan; 4 Department of Veterinary Medicine, National Pingtung University of Science and Technology, Pingtung, 91201, Taiwan; University of Bari, ITALY

## Abstract

Veterinary care plays an influential role in sea turtle rehabilitation, especially in endangered species. Physiological characteristics, hematological and plasma biochemistry profiles, are useful references for clinical management in animals, especially when animals are during the convalescence period. In this study, these factors associated with sea turtle surviving were analyzed. The blood samples were collected when sea turtles remained alive, and then animals were followed up for surviving status. The results indicated that significantly negative correlation was found between buoyancy disorders (BD) and sea turtle surviving (*p* < 0.05). Furthermore, non-surviving sea turtles had significantly higher levels of aspartate aminotranspherase (AST), creatinine kinase (CK), creatinine and uric acid (UA) than surviving sea turtles (all *p* < 0.05). After further analysis by multiple logistic regression model, only factors of BD, creatinine and UA were included in the equation for calculating summarized health index (SHI) for each individual. Through evaluation by receiver operating characteristic (ROC) curve, the result indicated that the area under curve was 0.920 ± 0.037, and a cut-off SHI value of 2.5244 showed 80.0% sensitivity and 86.7% specificity in predicting survival. Therefore, the developed SHI could be a useful index to evaluate health status of sea turtles and to improve veterinary care at rehabilitation facilities.

## Introduction

Veterinary care plays an influential role in sea turtle rehabilitation, especially in endangered species. Five species of sea turtles have been found around Taiwan, including green turtles (*Chelonia mydas*), hawksbill turtles (*Eretmochelys imbricate*), loggerhead turtles (*Caretta caretta*), olive ridley turtles (*Lepidochelys olivacea*), and leatherback turtles (*Dermochelys coriacea*) [1–2]. These turtle species are all included on the Red List of threatened species maintained by the World Conservation Union (IUCN Red List), and are also listed as endangered species under the Schedule of Protected Species by the Forestry Bureau, Council of Agriculture, Executive Yuan, Taiwan. In Taiwan, sea turtles that are stranded or accidentally trapped by fishing nets are routinely reported by the Coast Guard Administration, Executive Yuan, Taiwan. Following physiological examination, apparently healthy sea turtles are directly released into their natural environment. However, sea turtles suffering from traumatic injuries, emaciation, or other abnormalities are transported to a nearby rehabilitation facility for further medical care or long-term rehabilitation.

Health indices, such as physiological characteristics, hematological and plasma biochemistry profiles, are useful references for clinical management in animals, especially during the convalescence period [3–8]. Variations in plasma biochemistry and hematological values according to disease and physiological conditions have been reported for sea turtles [9–14]. For example, sick turtles were found to have significantly higher values of urea and aspartate aminotranspherase (AST) as well as lower packed cell volume (PCV) than healthy turtles [10]. Caliendo et al. [12] further reported that rehabilitated turtles had significantly higher PCV and a significantly lower number of white blood cells (WBC), heterophils, monocytes, AST, and creatinine kinases (CK). Non-surviving sea turtles have significantly greater plasma levels of sodium, chloride, potassium, calcium, phosphorus, and uric acid at the start of rehabilitation than do surviving sea turtles [15]. Nonetheless, reports investigating the differences in physiological characteristics, hematological and plasma biochemistry profiles between stranded sea turtles that ultimately survived and died are limited [7–8, 15].

This study compared the clinical characteristics, plasma biochemistry and hematological profiles between survival and non-survival sea turtles. After identification of individual predictive factors, a summary health index (SHI) based on the these factors was developed. The SHI could provide marine veterinarians an overall reference for use in predicting survival in sea turtles in rehabilitation facilities.

## Materials and Methods

### Study Subjects and Sample Collection

A total of 96 blood samples were collected from sea turtles during the period from 2011 to 2014. Sea turtles that appeared healthy were clinically examined and blood samples were collected in the field. Most of them were released back to their natural environment within 24 hrs. Unhealthy animals (i.e., emaciated, weak, or injured sea turtles) were transported to rehabilitation facilities under the authority of the Forestry Bureau, Council of Agriculture, Executive Yuan, Taiwan, where blood collection was performed after health assessment. After physical examination, these animals were monitored by endoscopy, radiography, serial blood profiles and plasma biochemical values in the rehabilitation facility. Other information, such as turtle species, date of rescue, possible cause of stranding, length of curved carapace and buoyancy disorders (BD), were also recorded by veterinarian for further statistical analysis.

### Examination of Hematological Values and Plasma Biochemistry Profiles

Approximately 12 ml of blood specimen was humanely collected by a certified veterinarian, from each animal via external jugular vein using a 12–15 ml syringe fitted with a 19–23 gauge needle after using 70% alcohol solution to disinfect the skin of puncture site. Whole blood was immediately placed in lithium heparin or buffered citrate sodium solution for further hematology and plasma biochemistry analyses. The blood samples were kept on ice until being processed. Plasma was separated from blood cells through 3,000 rpm centrifugation for 5 min before being transported to the Clinical Pathological Laboratory, National Pingtung University of Science and Technology, Pingtung, Taiwan, for analysis. If more than one blood sample was collected from the animal during the follow-up period, only the first specimen was used for analysis in this study.

Hematological examinations included analysis of packed cell volume (PCV), red blood cells (RBC), hemoglobin (HB), mean corpuscular volume (MCV), mean corpuscular hemoglobin (MCH), mean corpuscular hemoglobin concentration (MCHC), white blood cells (WBC) and, platelets (PLT), RBC counts and HB values were measured using a hematology analyzer (Mythic 18 Vet, BioVendor, Geneve, Switzerland). MCV, MCH, and MCHC were calculated using standard equations. PLT and WBC counts were performed with an automated hematology analyzer (Cell-Dyn 3700; Abbott, Santa Clara, California, USA). Differential counts were also manually performed on blood films to determine the percentage of total leukocytes and the absolute counts of heterophils, lymphocytes, monocytes, eosinophils, and basophils [16]. Blood smears were prepared from whole blood using lithium heparin and allowed to air dry. All smears were stained with Liu's stain. Leukocytes were categorized into one of five groups: heterophils, lymphocytes, monocytes, eosinophils, and basophils [17, 11].

Blood biochemistry profiles included total protein (TP), albumin (ALB), globulin (GLB), total bilirubin (TBIL), aspartate aminotransferase (AST), alanine aminotransferase (ALT), alkaline phosphatase (ALP), γ-glutamyltranspeptidase (GGT), creatinine kinase (CK), lactate dehydrogenase (LDH), cholesterol (CHOL), triglyceride (TRI), glucose (GLU), creatinine (CRE), blood urea nitrogen (BUN), uric acid (UA), phosphorous (PHOS), calcium (CA), sodium (NA), potassium (K), chloride (CL), lactic acid (LA) iron (FE), and fibrinogen (FIB). NA, K, and CL were processed on an ion-selective electrode analyzer (EasyLyte analyzer PLUS, Bedford, MA, USA). LA values were obtained using Kodak Ektachem DT-60 chemistry analyzers (Eastman Kodak Co., Rochester, New York, USA). Serum FE was measured on a Kodak Ektachem DT-60 chemistry analyzer with the DTSC II module (Johnson & Johnson Clinical Diagnostics, New York, USA). Plasma levels of TP, ALB, GLB, TBIL, AST, ALT, ALP, GGT,CK, LDH, CHOL, TRI, GLU, CRE, BUN, UA, CA, and PHOS were measured using the Fujifilm Dri-Chem 3500S system (Fujifilm, Tokyo, Japan). FIB concentration was determined using a Sysmex CA-500 automated coagulometer (TOA Medical Electronics Ltd, Co., Japan).

## Statistical Analysis

To develop a health index that could predict sea turtle surviving, data of turtle species, clinical characteristics, plasma biochemistry and hematological profiles (the data could be referred to S1 File listed in the supporting information) were compared between non-surviving (n = 11; *Chelonia mydas*: 8, *Lepidochelys olivacea*: 2; *Eretmochelys imbricatea*: 1) and surviving (n = 85; *Chelonia mydas*: 57, *Caretta caretta*: 12, *Lepidochelys olivacea*: 3; *Eretmochelys imbricate*: 12, *Dermochelys coriacea*: 1) sea turtles. Sea turtles that died within 4 months after the first blood collection were classified as non-surviving. The groups of surviving turtles were from two sources: (A) animals (n = 35) were accidentally caught by fishermen and were apparently healthy after gross examination. These animals were bled and released immediately after blood collection; (B) animals (n = 50) with unhealthy condition were delivered to a rehabilitation facility, where blood samples were collected after health assessment. These animals survived under veterinary care and released back to the sea after careful evaluation by veterinarians.

To determine which factors were associated with surviving, Mann-Whitney U-test or a t-test (depending on whether data fit normal distribution) was conducted to compare hematology and plasma biochemistry data between surviving and non-surviving turtle groups. Continuous data with normal distribution were expressed by mean ± standard deviation (SD) to show data variation, and non-normally distributed continuous data were reported by median and the limits of the overall range. To compare associations of categorical parameters that described physiological characteristics between the two groups, Fisher’s exact test was applied. For all tests, a *p* value less than 0.05 was considered statistically significant. To generate the best equation for predicting sea turtle surviving, the identified significant factors associated with survival were further analyzed by multiple logistic regression model. After using backward selection at the chosen critical level (*p* < 0.1), the best predictive equation was constructed and used to calculate logarithm of surviving odds for each animal. The logarithm of surviving odds was treated as the summarized health index (SHI) for each animal. The relationship of sea turtle surviving and SHI was evaluated by the area under the receiver operating characteristic curve (AUROC). Finally, using ROC curve, the most appropriate cut-off value of SHI to predict sea turtle surviving was determined by the point of SHI with maximum sum of sensitivity and specificity. All statistical analyses were performed by SPSS for Windows version 12.0 (Chicago, Illinois, USA).

## Ethics Statement

All blood samples were collected under humane procedures from an external jugular vein as described by Day et al. [13]. In this study, collection of sea turtle blood samples was from two main sources. One sample source was from the animals accidentally caught by fishermen during fishing work. These sea turtles were apparently healthy after physical examination and directly released to the sea after blood collection. In Taiwan, studies on endangered animal species, including sea turtles, need to be permitted by Forestry Bureau, Council of Agriculture, Executive Yuan, Taiwan. After getting the approval by Animal Care and Use Committee (IACUC) of National Taiwan Ocean University, Taiwan (Approval No:100050, 101050 and 102058; please refer to S2 File), the field work and the sampling method relevant to animal welfare issues were permitted under the protected wildlife use permissions of Forestry Bureau, Council of Agriculture, Executive Yuan, Taiwan (AF No.1001700596, AF No.1011700180, AF No.1021700541 and AF No.1031700537). The other sample source was from diseased sea turtles identified on seashores through the official reporting system. The animals were delivered to the rehabilitation facility at National Museum of Marine Biology and Aquarium (NMMBA), Pingtung, Taiwan, for further medical care. Therefore, the blood sample was necessarily collected by veterinarians for health examination without IACUC permission. The rescue work at NMMBA was authorized by Forestry Bureau, Council of Agriculture, Executive Yuan, Taiwan, through the funding support of 100 Forest-02.1-conserv-10(7), 101 Forest-02.1-conserv-10(7), 102 Forest-08.1-conserv-12 and 103 Forest -01.1-conserv-11(6). In this study, Drs. Tsungu-Hsien Li and I-Jiunn Cheng were responsible for blood sample collection.

## Results

The mean curved carapace length of animals in surviving and non-surviving groups was 62.7 ± 21.6 cm (range: 26.0–115.0 cm) and 60.1 ± 16.9 cm (range: 37.8–96.0 cm), respectively. Comparing to 9.4% (8/85) of sea turtles with BD in the surviving group, significantly higher percentage (72.7%) of animals with BD was observed in non-surviving group (*p*<0.05). No significant difference of barnacle infestation was observed between surviving and non-surviving groups (20.0% vs. 27.3%). Various hematological profiles were similar between surviving and non-surviving sea turtles (Table 1).

**Table 1:** The comparison of hematological profiles between surviving and non-surviving sea turtles.

Variable	Surviving Group (n = 85)	Non-surviving Group (n = 11)	*P* Value
PCV ^b^ (%)	32.52 (7.81)	36.00 (14.03)	0.485
RBC ^a^ (×10^6^/μl)	0.48 (0.26–1.62)	0.46 (0.16–1.42)	0.900
Hb ^b^ (g/dl)	9.86 (2.25)	10.11 (3.56)	0.835
MCV ^b^ (fl)	685.80 (138.35)	704.39 (158.27)	0.737
MCH ^b^ (pg)	207.76 (48.60)	204.89(59.28)	0.905
MCHC ^a^ (g/dl)	30.93 (18.60–47.80)	31.00 (20.00–33.60)	0.627
WBC ^a^ (μl^-1^)	9135.00 (1332.00–19900.00)	11160.00 (2900.00–79000.00)	0.730
Heterophils ^a^ (μl^-1^)	7372.00 (666.00–19104.00)	6338.00 (2639.00–46610.00)	0.949
Lymphocytes ^a^ (μl^-1^)	774.00 (0.00–9779.00)	927.00 (0.00–4262.00)	0.872
Monocytes ^a^ (μl^-1^)	386.00 (0.00–3219.00)	363.00 (0.00–31600.00)	0.616
Eosinophils ^a^ (μl^-1^)	0.00 (0.00–1760.00)	0.00 (0.00–188.00)	0.192
Thrombocytes ^a^ (×10^3^/μl)	12.60 (0.00–102.00)	11.46 (7.80–42.10)	0.909

^a^ Data were presented by median (the limit of all range) and compared by Mann–Whitney U test.

^b^ Data were presented by mean (standard deviation) and compared by Student’ s t-test.

The results of plasma biochemistry examinations were shown in Table 2. Non-surviving sea turtles had significantly higher AST, CK, creatinine, and UA values than surviving ones (all *p* < 0.05). It was also identified that sea turtles with BD were with higher AST, CK and UA than those without BD (all *p* < 0.05) (Table 3).

**Table 2:** The comparison of plasma biochemistry profiles between surviving and non-surviving sea turtles.

Variable	Surviving Group (n = 85)	Non-surviving Group (n = 11)	*P* Value
Total protein ^a^ (g/dl)	3.70 (1.60–7.80)	4.20 (1.50–7.40)	0.534
Albumin ^a^ (g/dl)	1.70 (0.70–2.70)	2.00 (0.80–3.60)	0.138
Globulin ^a^ (g/dl)	2.00 (0.70–5.70)	1.90 (0.60–4.70)	0.536
Total bilirubin ^a^ (mg/dl)	0.20 (0.10–0.80)	0.40 (0.10–4.30)	0.084
Aspartate aminotransferase ^a^ (U/L)	121.00 (11.80–1330.0)	271.00 (123.00–534.00)	0.001
Alanine aminotransferase ^a^ (U/L)	1.00 (1.00–83.00)	4.00 (1.00–30.00)	0.212
Alkaline phosphatase ^a^ (U/L)	36.00 (2.00–236.00)	26.00 (19.00–112.00)	0.344
γ-glutamyltranspeptidase ^a^ (U/L)	5.00 (1.00–19.00)	7.50 (1.00–62.00)	0.289
Creatinine kinase ^a^ (U/L)	1764.00 (116.00–13840.00)	2000.00 (966.00–25000.00)	0.029
Lactate dehydrogenase ^a^ (U/L)	264.00 (14.00–4464.00)	322.00 (84.00–8800.00)	0.485
Cholesterol ^a^ (mg/dl)	162.50 (44.00–450.00)	175.00 (55.00–450.00)	0.554
Triglyceride ^a^ (mg/dl)	46.00 (2.00–2526.00)	111.00 (20.00–306.00)	0.259
Glucose ^a^ (mg/dl)	110.00 (54.00–235.00)	101.00 (3.00–368.00)	0.482
Creatinine ^a^ (mg/dl)	0.10 (0.10–2.70)	0.40 (0.10–1.10)	0.007
Blood urea nitrogen ^a^ (mg/dl)	32.40 (0.60–140.00)	45.10 (7.00–117.70)	0.409
Uric acid ^a^ (mg/dl)	0.90 (0.30–9.90)	1.80 (0.70–6.40)	0.008
Phosphorus ^a^ (mg/dl)	8.10 (5.10–15.00)	7.80 (5.70–15.00)	0.778
Calcium ^a^ (mg/dl)	7.10 (0.10–13.80)	8.40 (2.70–14.00)	0.455
Sodium ^b^ (mEq/L)	148.41 (5.04)	148.54 (8.41)	0.945
Potassium ^a^ (mEq/L)	3.55(2.40–8.20)	3.72 (2.30–20.40)	0.509
Chloride ^b^ (mEq/L)	113.74 (5.45)	116.02 (6.90)	0.228
Lactate ^a^ (mg/dl)	4.40 (0.10–58.00)	1.65 (0.70–18.00)	0.699
Plasma iron ^a^ (μg/dl)	47.00 (10.00–407.00)	38.00 (10.00–407.00)	0.321
Fibrinogen ^a^ (mg/dl)	272.25 (73.80–900.00)	202.80 (88.00–900.00)	0.442

^a^ Data were presented by median (the limit of all range) and compared by Mann–Whitney U test.

^b^ Data were presented by mean (standard deviation) and compared by Student’s t-test.

**Table 3:** The comparison of plasma biochemical levels between sea turtles with and without buoyance disorder (BD).

Variable	With BD (n = 16)	Without BD (n = 80)	*P* Value
AST (U/L)	269.50 (61.00–588.00)	121.50 (11.80–1330.00)	0.019
CK (U/L)	2000.00 (271.00–25000.00)	1764.50 (116.00–13840.00)	0.057
Creatinine (mg/dl)	0.10 (0.10–1.10)	0.10 (0.10–2.70)	0.768
Uric acid (mg/dl)	1.85 (0.40–6.40)	0.90 (0.30–9.90)	0.007

^a^ Data were presented by median (the limit of all range) and compared by Mann–Whitney U test.

After univariate analsyis, significant plasma biochemistry parameters and BD were entered into a multiple logistic regression model to identify the best predictive model. After analysis by backward selection, only BD, creatinine and UA were incorporated in the final model to calculate SHI for each individual (Table 4). The developed equation was as following:

SHI = log (odds of surviving)=3.554*(BD)+1.306*(Creatinine level)+0.362*(UA level).

**Table 4:** The best predictive model determined by multiple logistic regression after backward selection.

Variable	aOR^a^ (95% CI^b^)	*P* Value
Buoyance Disorder (BD)	34.93 (5.65–215.88)	<0.001
Creatinine	3.69 (0.92–14.70)	0.06
Uric acid	1.43 (0.94–2.17)	0.08

^a^aOR: adjusted odds ratio.

^b^ CI: confidence interval.

**Fig 1:**
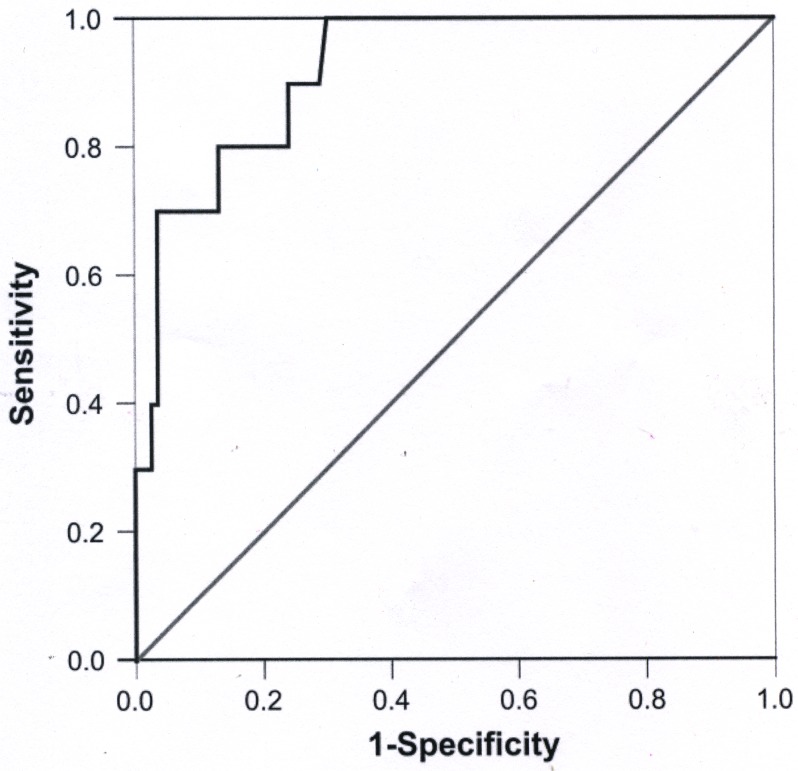
Evaluation of the summarized health index (SHI) for predicting sea turtle surviving by analysis of receiver operating characteristic (ROC) curve.

Here, when sea turtles showed BD, the value was one; otherwise, the value was zero. After evaluated by ROC analysis, the performance of SHI for surviving prediction was with AUROC of 0.920 ± 0.037 (Fig. 1). A cut-off SHI value of 2.5244 was with sensitivity of 80.0% and specificity of 86.7%.

## Discussion

Although previous studies have reported reference values of hematologic and plasma biochemistry values in sea turtles [18–20], most of them were limited by sample size and the values were with high variation possibly due to age and animal species. This is the first study to compare blood profiles and physiological features between surviving and non-surviving sea turtles. The blood samples were collected when sea turtles remained alive, and then animals were followed up for surviving status. Results from multiple logistic regression analysis indicated that only BD, plasma creatinine concentration and UA level were influential factors associated with sea turtle surviving. We further developed a summarized health index (SHI) as an overall health reference, and it was found that a cut-off SHI value of 2.5244 was with 80.0% sensitivity and 86.7% specificity for use in predicting sea turtle surviving.

It is important to note that hematological and plasma biochemical profiles could be different in various sea turtle species, and thus animal species may be influential to the estimated SHI. In this study, samples from five species of sea turtles were collected, including *Chelonia mydas*, *Caretta caretta*, *Lepidochelys olivacea*, *Eretmochelys imbricate*, *Eretmochelys imbricate* and *Dermochelys coriacea*. Most of the samples were from *Chelonia mydas*, and other species were with very limited sample size. Therefore, similar to the previous report [21], the limitation of this study is that the developed SHI cannot be further evaluated for each species. Nevertheless, no association of sea turtle species and surviving was identified in this study, though higher percentage of death (2/3; 66.7%) in *Lepidochelys olivacea*. Further comparison of blood profiles among sea turtle species by univariate analysis, it was identified that total bilirubin, aspartate aminotransferase, alkaline phosphatase, γ-glutamyltranspeptidase, lactate dehydrogenase, triglyceride, sodium and lactate were shown to be statistically different among various species (all *p <* 0.05). After model selection by multiple logistic regression analysis, these factors were not associated with survival and not included in SHI. Moreover, using only data from *Chelonia mydas* to validate SHI for predicting survival, the AUROC was 0.957 ± 0.029, and a cut-off SHI value of 3.7292 was with 85.7% sensitivity and 96.4% specificity. The result indicated that the developed SHI could be applied and worthy of further studies in various sea turtle species.

In this study, the surviving group included two different sources of sea turtles. One group of the animals were bled and released immediately after blood collection, because these animals were accidentally caught by fishermen and were apparently healthy after gross examination. It is hard to justify that these animals could be survived for more than 4 months. However, using only animals transferred to rehabilitation facilities to validate SHI, it revealed that the AUROC was 0.911 ± 0.040, and a cut-off SHI value of 4.0150 was with 80.0% sensitivity and 91.7% specificity, which again showed the use of SHI as a useful tool in sea turtle survival prediction.

Our results indicated that BD was highly and negatively associated with sea turtle surviving, perhaps due to an inability to dive and forage for food. However, many other health conditions were associated with BD, and the main reasons causing BD in sea turtles were poorly understood [22]. Most cases of sea turtles with BD have been attributed to lung tears, pneumonia, intestinal impactions, neurologic damage, or to the effects of being cold-stunned [22–23]. However, a study on stranded green sea turtles (*Chelonia mydas*), reported that gastrointestinal or respiratory disorders were not significantly associated with BD [24]. In our study, turtles exhibiting symptoms of BD were found to have significantly higher concentrations of AST and UA, comparing to turtles without BD. Elevated AST and UA levels suggest that sea turtles with BD may suffer from dehydration, decreased renal function, damage to the liver, or injured skeletal muscles [4, 25–26]. These factors themselves were also the possible causes of death. Therefore, whether BD itself an independent factor causing death or playing an intermediated role on the death pathway needs to be further elucidated in the future. However, it is of major importance to include BD in SHI for offering early special care in rescued sea turtles, as a careful supportive therapy and together with the treatment of the cause could be very helpful to solve BD.

Due to low level of plasma creatinine produced by reptiles (<1 mg/dl), this substance is generally considered to have no diagnostic value clinically [4, 15, 27–28]. In this study, plasma creatinine level in surviving turtles was similar to that in nesting leatherback sea turtles (*Dermochelys coriacea*) as previously reported [29], and that in clinically healthy green sea turtles (*Chelonia mydas*) [1]. We further observed significantly higher plasma creatinine level in sea turtles that eventually died than that in surviving ones. In other vertebrate animals, higher plasma creatinine level is associated with renal function, which could affect survivorship [30–31]. Renal failure could be one of the factors causing death. Although limited studies have been reported in sea turtles, Knotek et al. [32] found higher creatinine levels in green iguanas (*Iguana iguana*) with renal disease than those in healthy green iguanas. It suggests that concentration of plasma creatinine may be applied for diagnosis of renal failure in reptiles. In our study, of major importance, based on the results by both univariate and multiple logistic regression analyses, it was highly supported that the plasma creatinine concentration could be used as the predictive factor of sea turtle surviving.

The UA level has been recommended as the best indicator of nephropathy in reptiles [4]. We found that the mean UA value in non-surviving sea turtles was higher than that in surviving ones, and also higher than that in nesting hawksbill turtles (*Eretmochelys imbricata*) reported by Goldberg et al. [33]. In previous research on hematologic and plasma biochemistry among Kemp’s Ridley sea turtles (*Lepidochelys kempii*) suffering from cold stunning, UA level in the non-surviving group was significantly higher than that in the surviving group [15]. It was found that UA level in green sea turtles affected by cold stunning was slightly higher than in turtles with normal physiological conditions [6]. Deem et al. [25] further documented that the UA values in stranded green sea turtles were significantly higher than those of nesting and foraging turtles, and the higher UA value may result from debilitation and dehydration.

In our study, although AST and CK levels were associated with sea turtle surviving by univariate analysis, these two factors were not included in the final equation of SHI after evaluated by multiple logistic regression, possibly due to the high association of these two factors with BD. Clinically, elevated AST levels were related to liver damage, skeletal muscle damage, or cardiac muscle disorders [4, 26], indicating that AST is not organ specific [4, 34]. The AST value in fully rehabilitated hawksbill turtles was significantly lower than that in turtles undergoing convalescence [12]. Higher AST values have also been reported in green sea turtles with fibropapillomatosis [10, 35]. On the other hand, the CK level in non-surviving sea turtles was higher than that in surviving ones. Damages of skeletal muscles in reptiles, frequently observed in animals struggling to resist restraint, could result in elevated concentration of CK [4]. In leatherback (*Dermochelys coriacea*) and loggerhead sea turtles (*Caretta caretta*), higher CK level was observed in animals with obvious muscle injuries or wasting conditions [27, 29–30]. Moreover, increase of CK concentration due to intramuscular injection has been reported in reptiles [4]. The previous study found that heart and arteries are the primary sites after spirorchiid infection in sea turtles, and damages of these muscles by the infection may also increase CK level [36].

Our study identified significant differences of BD, plasma creatinine concentration and UA level between surviving and non-surviving sea turtles. The developed SHI, which was calculated by the level of these factors, may provide marine veterinarians for health evaluation in sea turtles in wildlife rehabilitation facilities. The SHI could be applied for prediction of sea turtle surviving after stranding, and offer timely and more appropriate clinical cares.

## Supporting Information

S1 File S1_Dataset.xls was used for the analysis of this study. (XLS)

S2 File IACUC ethics approval documents. (PDF)

## References

[1] Fong CL, Chen HC, Cheng IJ (2010) Blood profiles from wild populations of green sea turtles in Taiwan. Journal of Veterinary Medicine and Animal Health 2: 8–10.

[2] King R, Cheng W-H, Tseng C-T, Chen H, Cheng I-J (2013) Estimating the sex ratio of green sea turtles (Chelonia mydas) in Taiwan by the nest temperature and histological methods. Journal of Experimental Marine Biology and Ecology 445: 140–147.

[3] Lutcavage ME, Lutz PL, Bossart GD, Hudson DM (1995) Physiologic and Clinicopathologic Effects of Crude Oil on Loggerhead Sea Turtles. Archives of Environmental Contamination and Toxicology 28: 417–422. 7755395 10.1007/BF00211622

[4] Campbell TW (2006) Clinical pathology of reptiles In: Reptile medicine and surgery, 2nd ed. Mader DR editors. St. Louis, Missouri: Saunders Elsevier pp. 453–470.

[5] Flint M, Morton JM, Limpus CJ, Patterson-Kane JC, Mills PC (2010a) Reference intervals for plasma biochemical and hematologic measures in loggerhead sea turtles (Caretta caretta) from Moreton Bay, Australia. Journal of Wildlife Diseases 46: 731–741. 20688679 10.7589/0090-3558-46.3.731

[6] Anderson ET, Harms CA, Stringer EM, Cluse WM (2011) Evaluation of Hematology and Serum Biochemistry of Cold-Stunned Green Sea Turtles (Chelonia mydas) in North Carolina,USA. Journal of Zoo and Wildlife Medicine 42: 247–255. 22946402 10.1638/2010-0217.1

[7] Keller KA, Innis CJ, Tlusty MF, Kennedy AE, Bean SB, Cavin JM, et al (2012) Metabolic and respiratory derangements associated with death in cold-stunned Kemp’s ridley turtles (Lepidochelys kempii): 32 cases (2005–2009). Journal of the American Veterinary Medical Association 240: 317–323. 10.2460/javma.240.3.317 22256849

[8] Camacho M, Quintana MP, Luzardo OP, Estévez MD, Calabuig P, Orós J, et al (2013b) Metabolic and respiratory status of stranded juvenile loggerhead sea turtles (Caretta caretta):66 cases (2008–2009). Journal of the American Veterinary Medical Association 242: 396–401. 10.2460/javma.242.3.396 23327184

[9] Santoro M, Meneses A (2007) Haematology and plasma chemistry of breeding olive ridley sea turtles (Lepidochelys olivacea). Veterinary Record 161: 818–819. 18083982

[10] Whiting SD, Guinea ML, Limpus CJ, Fomiatti K (2007) Blood chemistry reference values for two ecologically distinct population of foraging green turtles, eastern Indian Ocean. Comparative Clinical Pathology 16: 109–118.

[11] Casal AB, ORóS J (2007) Morphologic and cytochemical characteristics of blood cells of juvenile loggerhead sea turtles (Caretta caretta). Research in Veterinary Science 82: 158–165. 17067648 10.1016/j.rvsc.2006.07.017

[12] Caliendo V, McKinney P, Robinson D, Baverstock W, Hyland K (2010) Plasma biochemistry and hematology values in juvenile hawksbill turtles (Eretmochelys imbricata) undergoing rehabilitation. Journal of Herpetological Medicine and Surgery 20: 117–121.

[13] Day RD, Keller JM, Harms CA, Segars AL, Cluse WM, Godfrey MH, et al (2010) Comparison of mercury burdens in chronically debilitated and healthy loggerhead sea turtles (Caretta Caretta). Journal of Wildlife Diseases 46: 111–117. 20090024 10.7589/0090-3558-46.1.111

[14] Delgado C, Valente A, Quaresma I, Costa M, Dellinger T (2011) Blood biochemistry reference values for wild juvenile loggerhead sea turtles (Caretta caretta) from madeira archipelago. Journal of Wildlife Diseases 47: 529–529. 10.7589/0090-3558-47.3.523 21719817

[15] Innis CJ, Ravich JB, Tlusty MF, Hoge MS, Wunn DS, Boerner-Neville LB, et al (2009) Hematologic and plasma biochemical findings in cold-stunned Kemp’s Ridley turtles: 176 cases (2001–2005). Journal of the American Veterinary Medical Association 235: 426–432. 10.2460/javma.235.4.426 19681727

[16] Stamper MA, Harms C, Epperly SP, Braunmcneill J, Avens L, Stoskopf MK, et al (2005) Relationship between barnacle epibiotic load and hematologic parameters in loggerhead sea turtles (Caretta caretta): A comparison between migratory and residential animals in Pamlico Sound, North Carolina. Journal of Zoo and Wildlife Medicine 36: 635–641. 17312720 10.1638/04-074.1

[17] Work TM, Raskin RE, Balazs GH, Whittaker SD (1998) Morphological and cytochemical characteristics of blood cells from Hawaiian green turtles. American Journal of Veterinary Research 59: 1252–1257. 9781457

[18] Casal AB, Camacho M, López-Jurado LF, Juste C, Orós J (2009) Comparative study of hematologic and plasma biochemical variables in Eastern Atlantic juvenile and adult nesting loggerhead sea turtles (Caretta caretta). Veterinary Clinical Pathology 38: 213–218. 10.1111/j.1939-165X.2008.00106.x 19192261

[19] Camacho M, Luzardo OP, Boada L D, López Jurado LF, Medina M, Zumbado M, et al (2013a) Potential adverse health effects of persistent organic pollutants on sea turtles: Evidences from a cross-sectional study on Cape Verde loggerhead sea turtles. Science of the Total Environment 458–460: 283–289. 10.1016/j.scitotenv.2013.04.043 23665416

[20] Lewbart GA, Hirschfeld M, Denkinger J, Vasco K, Guevara N, Garcia J, et al (2014) Blood Gases, Biochemistry, and Hematology of Galapagos Green Turtles (Chelonia Mydas). PLoS One 9: e96487 10.1371/journal.pone.0096487 24824065 PMC4019482

[21] Wolf KN, Harma CA, Beasley JF (2008) Evaluation of five clinical chemistry analyzers for use in health assessment in sea turtles. Journal of the American Veterinary Medical Association 233: 470–475. 10.2460/javma.233.3.470 18673037

[22] Schmitt TL, Munns S, Adams L, Hicks J (2013) The use of spirometry to evaluate pulmonary function in Olive Ridley Sea Turtles (Lepidochelys Olivacea) with Positive Buoyancy Disorders. Journal of Zoo and Wildlife medicine 44: 645–653. 24063092 10.1638/2012-0210R.1

[23] Wyneken J, Mader DR, Webber ES 3rd, Merigo C (2006) Medical management of sea turtles In: Mader DR editor. Reptile Medicine and Surgery. St. Louis, Missouri: Saunders Elsevier pp. 972–1007.

[24] Flint M, Patterson-Kane JC, Limpus CJ, Mills PC (2010b) Health surveillance in stranded green sea turtles in southern Queensland, Australia (2006–2009): an epidemiological analysis of the causes of disease and mortality in marine turtles. EcoHealth 7: 135–145. 10.1007/s10393-010-0300-7 20232226

[25] Deem SL, Norton TM, Mitchell M, Segars A, Alleman AR, Cray C, et al (2009) Comparison of blood values in foraging, nesting, and stranded loggerhead turtles (Caretta caretta) along the coast of Georgia, USA. Journal of Wildlife Diseases 45: 41–56. 19204334 10.7589/0090-3558-45.1.41

[26] Harris HS, Benson SR, Gilardi KV, Poppenga RH, Work TM, Dutton PH, et al (2011) Comparative health assessment of western Pacific leatherback turtles (Dermochelys coriacea) foraging off the coast of California: 2005–2007. Journal of Wildlife Diseases 47: 321–337. 21441185 10.7589/0090-3558-47.2.321

[27] Perrault JR, Miller DL, Eads E, Johnson C, Merrill A, Thompson LJ, et al (2012) Maternal health status correlates with nest success of leatherback sea turtles (Dermochelys coriacea) from Florida. PLoS One 7: e31841 10.1371/journal.pone.0031841 22359635 PMC3281022

[28] Rousselet E, Stacy NI, LaVictoire K, Higgins BM, Tocidlowski ME, Flanagan JP, et al (2013) Hematology and plasma biochemistry analytes in five age groups of immature, captive-reared loggerhead sea turtles (caretta caretta). Journal of Zoo and Wildlife Medicine 44: 859–874. 24450044 10.1638/2012-0162R1.1

[29] Deem SL, Dierenfeld ES, Sounguet GP, Alleman AR, Cray C, Poppenga RH, et al (2006) Blood values in free-ranging nesting leatherback sea turtles (Dermochelys coriacea) on the coast of the Republic of Gabon. Journal of Zoo and Wildlife Medicine 37: 464–471. 17315430 10.1638/05-102.1

[30] Jacob F, Polzin DJ, Osborne CA, Neaton JD, Kirk CA, Allen TA, et al (2005) Evaluation of the association between initial proteinuria and morbidity rate or death in dogs with naturally occurring chronic renal failure. Journal of the American Veterinary Medical Association 226: 393–400. 15702689 10.2460/javma.2005.226.393

[31] McLeland SM, Lunn KF, Duncan CG, Refsal KR, Quimby JM (2014) Relationship among serum creatinine, serum gastrin, calcium-phosphorus product, and uremic gastropathy in cats with chronic kidney disease. Journal of Veterinary Internal Medicine 28: 827–837. 10.1111/jvim.12342 24628683 PMC4895456

[32] Knotek Z, Hauptman K, Knotkov á Z, H ájkov á P, Tich ý F (2002) Renal disease haemogram and plasma biochemistry in green iguana. Acta Veterinaria Brno 71: 333–340.

[33] Goldberg DW, Leitão SAT, Godfrey MH, Lopez GG, Santos AJB, Neves FA, et al (2013) Ghrelin and leptin modulate the feeding behaviour of the hawksbill turtle Eretmochelys imbricata during nesting season. Conservation Physiology 1: 10.1093/conphys/cot016 PMC4732442 27293600

[34] Honarvar S, Brodsky MC, Fitzgerald DB, Rosenthal KL, Hearn GW (2011) Changes in plasma chemistry and reproductive output of nesting leatherbacks. Herpetologica 67: 222–235.

[35] Swimmer Y (2000) Biochemical responses to fibropapilloma and captivity in the green turtle. Journal of Wildlife Diseases 36: 102–110. 10682751 10.7589/0090-3558-36.1.102

[36] Chen H, Kuo RJ, Chang TC, Hus CK, Bray RA, Cheng IJ, et al (2012) Fluke (Spirorchiidae) infections in sea turtles stranded on Taiwan: prevalence and pathology. Journal of Parasitology 98: 437–439. 10.1645/GE-2875.1 22032290

