# Supplementary material for: Development of a Summarized Health Index (SHI) for Use in Predicting Survival in Sea Turtles
Source: PLoS One. 2015 Mar 24;10(3):e0120796. doi: 10.1371/journal.pone.0120796 (PMC4372459; doi:10.1371/journal.pone.0120796)
Supplement: S2 File — (PDF) [file pone.0120796.s002.pdf]

國立臺灣海洋大學生命科學院實驗動物照護及使用委員會審查同意書
Affidavit of Approval of Animal Use Protocol
College of Life Sciences, NTOU

動物實驗申請表暨同意書編號：100050

計畫申請人：程一驟　職稱：教授
單位：海洋生物所　飼養/應用地點：無須飼養/澎湖、蘭嶼、琉球
計畫名稱：1、小琉球海龜生態調查及保育規劃計畫
2、台東縣海龜生殖生態學暨保育研究計畫
3、澎湖附近海域海龜群族生態調查及推動伙伴關係

本計畫之「動物實驗申請表」業經實驗動物照護及使用委員會 ■實質□形式審查通過。本計畫預定飼養應用之動物如下：

動物種類	動物數量	計畫執行期間
綠蠵龜	30 隻	101 年 01 月 01 日至 101 年 12 月 31 日
赤蠵龜	10 隻	101 年 01 月 01 日至 101 年 12 月 31 日
玳瑁	10 隻	101 年 01 月 01 日至 101 年 12 月 31 日
欖蠵龜	10 隻	101 年 01 月 01 日至 101 年 12 月 31 日

The animal use protocol listed below has been reviewed and approved by the Institutional Animal Care and Use Committee（IACUC）.

Protocol Title：
ACUC Approval No：100050
Period of Protocol：Valid From: 01/01/2012 To: 12/31/2012 （mm/dd/yyyy）
Principle Investigator（PI）：I-J Cheng

國立臺灣海洋大學生命科學院

實驗動物照護及使用委員會主任委員 龔瑞林　日期 100.12.30
IACUC Chairman Zwe-Ling Kong　Date 12.30.2011

國立臺灣海洋大學生命科學院實驗動物照護及使用委員會審查同意書

Affidavit of Approval of Animal Use Protocol

College of Life Sciences, NTOU

動物實驗申請表暨同意書編號： 101050

計畫申請人： 程一駿 職稱： 教授

單位 ： 海洋生物所 飼養/應用地點：無須飼養/澎湖、台東、小琉球

計畫名稱 ：(1)澎湖縣海龜族群量生態調查及保護區經營管理計畫

(2)台東縣海龜生殖生態學暨保育研究計畫

(3)屏東縣琉球鄉綠蠵龜復育計畫

本計畫之「動物實驗申請表」業經實驗動物照護及使用委員會 ■實質□形式審查通過。本計畫預定飼養應用之動物如下：

動物種類	動物數量	計畫執行期間
綠蠵龜	60 隻	102 年 01 月 01 日至 102 年 12 月 31 日
赤蠵龜	10 隻	102 年 01 月 01 日至 102 年 12 月 31 日
玳瑁	10 隻	102 年 01 月 01 日至 102 年 12 月 31 日
欖蠵龜	10 隻	102 年 01 月 01 日至 102 年 12 月 31 日

The animal use protocol listed below has been reviewed and approved by the Institutional Animal Care and Use Committee（IACUC）.

Protocol Title：

ACUC Approval No： 101050

Period of Protocol： Valid From: 01/01/2013 To: 12/31/2013 （mm/dd/yyyy）

Principle Investigator（PI）： Cheng, I-Jiun

實驗動物照護及使用委員會主任委員 龔瑞林 日期 101.12.26

IACUC Chairman Zwe-Ling Kong Date 12.26.2012

國立臺灣海洋大學 生命科學院

國立臺灣海洋大學生命科學院實驗動物照護及使用委員會審查同意書
Affidavit of Approval of Animal Use Protocol
College of Life Sciences, NTOU

動物實驗申請表暨同意書編號：102058

計畫申請人：程一駿　職稱：教授
單位：海洋生物所
飼養/應用地點：實驗室、貢寮海龜救傷中心/澎湖、花蓮、臺東、新北市、宜蘭、苗栗、新竹、桃園、基隆市
計畫名稱：(1) 以生物紀錄儀配合洋流資訊來了解海龜洄游行為與海況間的關係(II)
(2) 復育基隆海岸生物多樣性-基隆地區近岸海洋生物多性評估及海洋生態復育重點規劃-基隆地區海域之底棲無脊椎動物群聚時空變化評析(1/3)
(3) 建立台東地區海龜保育策略及國際保育合作機制

本計畫之「動物實驗申請表」業經實驗動物照護及使用委員會 ■實質□形式審查通過。本計畫預定飼養應用之動物如下：

動物種類	動物數量	計畫執行期間
綠蠵龜(二級保育類)	60 隻/年	103年01月01日至103年12月31日
赤蠵龜(二級保育類)	10 隻/年	103年01月01日至103年12月31日
玳瑁(二級保育類)	10 隻/年	103年01月01日至103年12月31日
欖蠵龜(二級保育類)	10 隻/年	103年01月01日至103年12月31日

The animal use protocol listed below has been reviewed and approved by the Institutional Animal Care and Use Committee（IACUC）.

Protocol Title：

ACUC Approval No： 102058

Period of Protocol： Valid From: 01/01/2014 To: 12/31/2014 （mm/dd/yyyy）

Principle Investigator（PI）： Cheng, I-Jiunn

實驗動物照護及使用委員會主任委員 吳彰哲 日期 102.12.19

IACUC Chairman Chang-Jer Wu Date 12.19.2013

國立臺灣海洋大學 生命科學院
